# Macropinocytosis as a potential mechanism driving neurotropism of *Cryptococcus neoformans*

**DOI:** 10.3389/fcimb.2023.1331429

**Published:** 2023-12-11

**Authors:** Dylan M. Lanser, Amelia B. Bennett, Kiem Vu, Angie Gelli

**Affiliations:** Department of Pharmacology, School of Medicine, University of California, Davis, Davis, CA, United States

**Keywords:** macropinocytosis, EphA2, Ephrin receptor, EphrinA1, CD44, transcytosis, blood-brain barrier, brain endothelial cells

## Abstract

*Cryptococcus neoformans* can invade the central nervous system by crossing the blood-brain barrier via a transcellular mechanism that relies on multiple host factors. In this narrative, we review the evidence that a direct interplay between *C. neoformans* and brain endothelial cells forms the basis for invasion and transmigration across the brain endothelium. Adherence and internalization of *C. neoformans* is dependent on transmembrane proteins, including a hyaluronic acid receptor and an ephrin receptor tyrosine kinase. We consider the role of EphA2 in facilitating the invasion of the central nervous system by *C. neoformans* and highlight experimental evidence supporting macropinocytosis as a potential mechanism of internalization and transcytosis. How macropinocytosis might be conclusively demonstrated in the context of *C. neoformans* is also discussed.

## The pathophysiology of *Cryptococcal* neuro-infection

Diseases caused by fungal pathogens affect over a billion people and kill approximately 1.7 million annually (Bongomin et al., 2017). The severity of fungal disease varies from asymptomatic in healthy hosts to disseminated, life-threatening infections in immunosuppressed individuals. When compared to other infections of the central nervous system (CNS), infections caused by fungi have the highest morbidity and mortality. The primary reasons are that it is nearly impossible to completely eradicate the fungus from the brain and these infections tend to occur in individuals that are highly immunosuppressed, including HIV positive patients, organ transplant recipients and patients undergoing cancer treatments (Williamson et al., 2017).

Among fungal pathogens that can breach the CNS, *Cryptococcus neoformans* (*Cn*) is the most prevalent cause of adult brain fungal infection (Bongomin et al., 2017; Rajasingham et al., 2017; Rajasingham et al., 2022). *Cryptococcal meningitis* (CM) is the typical clinical presentation and cause of death during cryptococcosis (Mitchell and Perfect, 1995; Gottfredsson and Perfect, 2000). Without rapid intervention, CM is uniformly fatal regardless of the immune status of the host. Once inhaled, spores/desiccated yeast of *Cn* initially proliferate in lung tissue and eventually disseminate to the blood and then the CNS (Ellis and Pfeiffer, 1990; Velagapudi et al., 2009; Walsh et al., 2019), reviewed in (Chen et al., 2022). While dissemination can occur in any tissue, the CNS is the major site of invasion and growth of cryptococci. After breaching the blood-brain barrier (BBB), *Cn* forms self-contained cystic lesions referred to as cryptococcomas, where fungal cells can proliferate and damage the brain (Kwon-Chung et al., 2000; Park et al., 2009). Brain pathology consists of early cerebral vessel damage followed by fungal proliferation, first in the perivascular spaces and then in the deeper layer of the brain parenchyma with secondary seeding of the leptomeninges. Neurologic sequelae including visual loss, cranial palsies, neurologic deficit and mental impairment occurs in 40-50% of treated patients and 20-25% experience a relapsing course (Traino et al., 2019). Treatment guidelines for HIV-associated CM recommend amphotericin B with flucytosine for greater than 2 weeks as induction therapy followed by a triazole for a minimum of 10 weeks (Perfect et al., 2010). Fifty percent of AIDS patients treated for CM will experience a relapse of the disease unless they receive maintenance therapy. Unfortunately, in Africa and Asia, where disease burden is the highest, flucytosine treatment is prohibitively expensive (Loyse et al., 2013a; Loyse et al., 2013b). Despite the recent increase in antiretroviral therapy (ART) reducing the immunocompromised population in resource-deprived regions, the incidence of CM remains high (Pyrgos et al., 2013; Muzazu et al., 2022).

## Neuroinvasion of *Cn* and the role of the blood-brain barrier

**Figure 1:**
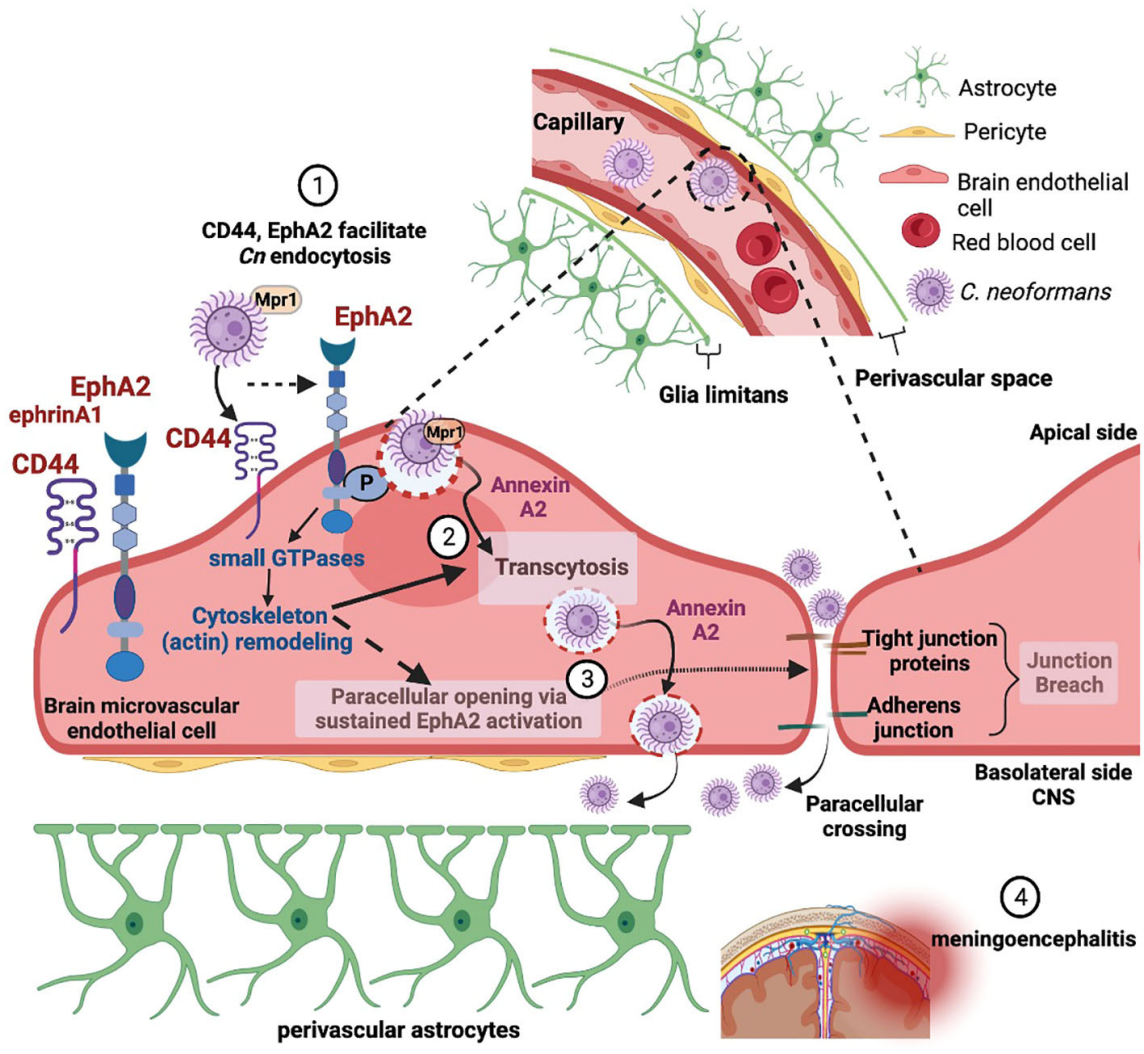
Adherence and internalization of *C. neoformans* depends on the (1) hyaluronic acid receptor (CD44) and an ephrin receptor tyrosine kinase (EphA2) - triggering membrane and cytoskeleton remodeling (2). EphA2-mediated signaling promotes macropinocytic transcytosis and opens a paracellular path via the re-modeling of tight junctions (3), thus facilitating invasion of the central nervous system by *C. neoformans* (4). Created with Biorender.com.

Recent studies have focused on the underlying molecular mechanisms facilitating the translocation of fungi from blood-to-brain along with the structural and molecular changes in the neurovascular unit (NVU) and how that informs neurological changes in the brain. Diffusion of bloodborne molecules across the BBB is highly restricted due to specialized endothelial cells that line the lumen of the brain microvasculature and the tight junction proteins in between endothelial cells that restrict paracellular flow (Keaney and Campbell, 2015). The BBB is part of the larger NVU, including pericytes and perivascular astrocytes that further support BBB function (Zheng, 2001; Weiss et al., 2009). Real-time imaging of mice after inoculation with *Cn* revealed that translocation of *Cn* from blood-to-brain begins with capillary sequestration, followed by a direct association between fungal cells and the endothelial surface of the capillary (Shi et al., 2010). This internalization and migration of *Cn* across the BBB was further confirmed *in vivo* with a flow cytometry approach that quantified *Cn* migration into the brain (Chen et al., 2021). This entire process can occur without involvement of macrophages or the breakdown of junctional proteins, suggesting that *Cn* can interact directly with proteins on the surface of brain endothelial cells (Kozel and Gotschlich, 1982; Ibrahim et al., 1995; Chang et al., 2004; Vu et al., 2009; Huang et al., 2011). However, other studies have clearly demonstrated the presence of the Trojan horse pathway, where *Cn* resides within host phagocytes on their way to the CNS during the course of infections (Charlier et al., 2009; Sorrell et al., 2016; Santiago-Tirado et al., 2017; Gaylord et al., 2020). The relative contribution to CNS invasion between transcellular crossing, where *Cn* is first internalized and then eventually exocytosed on the abluminal surface, and the Trojan horse pathway still remains to be resolved (**Figure 1**).

## Mechanisms facilitating *Cn* across the blood-brain barrier

Although observations of fungal cells in the CNS have been reported in real-time and at autopsy, the molecular interactions at the brain endothelium or the pathways that mediate the movement of fungal cells into the CNS do not yet form a coherent picture. Early studies using an *in vitro* BBB model system have shown that when *Cn* contacts the brain endothelium, the endothelial cells form membrane ruffles and cup-like F-actin structures (Chen et al., 2003; Chang et al., 2004). The surface changes are indicative of membrane and cytoskeleton re-modeling – alterations required for *Cn* to breach the BBB (Chen et al., 2003). Cytoskeletal rearrangements may eventually lead to altered permeability of the BBB via tight junction disruption, and *Cn* internalization itself may cause an upregulation in endocytic vesicle formation. Several studies have confirmed that the translocation of *Cn* from blood to the brain begins with brain microcapillary sequestration and involves a coordination of several virulence factors including phospholipase B1 (PLB1) (Maruvada et al., 2012), a metalloprotease (Mpr1) (Vu et al., 2014), urease (Olszewski et al., 2004), laccase (Qiu et al., 2012), a serine protease (Xu et al., 2014) and the host receptor, CD44 (Jong et al., 2008b; Jong et al., 2012),

## CD44

CD44 is critical for *Cn* adherence and invasion through an actin-dependent process of brain endothelial cells (Chang et al., 2006; Jong et al., 2007b; Jong et al., 2008a; Jong et al., 2008b; Jong et al., 2012) (**Figure 1**). CD44 functions as a receptor and anchor for hyaluronic acid (HA), a macromolecule that serves many roles, including as a component of the extracellular matrix of blood vessels and the capsule of *Cn* (Chen et al., 2021). *Cn* adheres to CD44 via hyaluronic acid on lipid rafts immediately prior to internalization (Huang et al., 2011). Perhaps analogously, lipid rafts form on brain endothelial cells prior to endocytosis of neuroinvasive *Escherichia coli* (Loh et al., 2017). The variant of CD44 expressed on brain endothelial cells appears to be the standard variant (Jong et al., 2008b), and therefore, *Cn* neurotropism cannot be a consequence of selective binding to a particular CD44 isoform expressed in brain microvasculature. CD44 is also not upregulated upon *Cn* exposure (Jong et al., 2008b). Nevertheless, acquisition of inositol, an abundant sugar in the brain, by *Cn* increases the expression of hyaluronic acid synthase (*CPS1*) and leads to greater production of HA which would promote adherence and stimulate internalization (Liu et al., 2013). Moreover, without hyaluronic acid synthase (*CPS1*), *Cn* neither produces a capsule nor invades the CNS (Chang et al., 2006; Jong et al., 2007).

## The Ephrin receptor tyrosine kinase, EphA2

Although hyaluronic-acid-CD44 interaction plays an early role in *Cn* invasion, there are other factors and/or processes necessary for *Cn* endocytosis (Maruvada et al., 2012; Vu et al., 2014; Na Pombejra et al., 2017). Transcriptomic analysis of brain endothelial cells exposed to *Cn* revealed increased expression of ephrinA1 (*EFNA1*), the ligand for the receptor tyrosine kinase EphA2 (Aaron et al., 2018). EphA2 belongs to the Eph A class of the Ephrin family of receptor tyrosine kinases (RTK) and along with their ligands (Ephrins) they make up the largest RTK subfamily (Himanen and Nikolov, 2003). Upregulation of ephrinA1 expression in endothelial cells has also been reported in response to inflammation (Funk et al., 2012), serum depletion (Wiedemann et al., 2017) ischemia (Chen et al., 2018a) and increased cell density (Wiedemann et al., 2017). The EphrinA1 ligand is membrane-tethered by a GPI linkage that subsequently binds to and activates the Eph A class of receptors (i.e., EphA2). Once activated, EphA2 receptors oligomerize and become autophosphorylated at juxtamembrane tyrosine residues.

In the case of *Cn*, silencing the EphA2 transcript in brain endothelial cells or inhibiting EphA2 activity with an antibody or an inhibitor (dasatinib) prevents *Cn* from crossing the brain endothelium in an *in vitro* model of the BBB, whereas treatment with recombinant ephrin A1 or an agonist enhances *Cn* crossing (Aaron et al., 2018). The inability of the *CPS1* deletion strain to induce the phosphorylation of EphA2 or to cross the BBB upon stimulating EphA2 with ephrinA1 supports the involvement of CD44 in the internalization *Cn* and suggests a connection between these host-expressed transmembrane proteins in the context of *Cn* invasion (Aaron et al., 2018). Interestingly, Kaposi’s sarcoma-associated herpesvirus (Hahn et al., 2012), *Chlamydia trachomatis* (Subbarayal et al., 2015), *Plasmodium falciparum* (Kaushansky et al., 2015; Darling et al., 2020), *Helicobacter pylori* (Leite et al., 2020), Epstein-Barr virus (Chen et al., 2018b) and uropathogens (Prakash et al., 2022) also enter host cells via EphA2.

Why or how *Cn* engages EphA2 is still under investigation, however the role of EphA2 in cytoskeleton remodeling (Pitulescu and Adams, 2010), in triggering signaling cascades of macropinocytosis (Hahn et al., 2012; Bandyopadhyay et al., 2014), and its influence on the integrity of the brain endothelium (Tanaka et al., 2005; Fang et al., 2008; Zhou et al., 2011) would suggest that *Cn* may be exploiting EphA2 for invasion of the CNS (**Figure 1**). Consistent with this notion, host factors (PI3K, MAPK, Src kinases, Rac1 and Rho- GTPases) activated by *Cn* to induce transcytosis across the BBB are known to be triggered by EphA2 for endocytosis (Jong et al., 2008a; Kim et al., 2012; Maruvada et al., 2012).

## Macropinocytosis

Macropinocytosis is a form of endocytosis defined by large endocytic vesicles and non-specificity (Salloum et al., 2023). Macropinosomes form from membrane protrusions supported by polymerizing actin; these protrusions extend then collapse inward, entrapping an extracellular volume within macropinosomes of 0.5-10 µm in diameter (Lin et al., 2020). Macropinocytosis is “macro” in the sense that the macropinosomes can be easily observed with traditional light microscopy, and exploited for cellular uptake by bacteria, larger pathogens, and protein aggregates, in contrast to other forms of pinocytosis (Mercer and Helenius, 2009; Bloomfield and Kay, 2016; Yerbury, 2016). Although the triggers leading to macropinocytosis are extremely diverse, there is some commonality in their dependence on Rac1 (Grimmer et al., 2002; Salloum et al., 2023). Macropinocytosis is common throughout Eukarya (Lin et al., 2020), including many human cell types. This form of endocytosis is particularly pronounced and well-studied in immune cells, with an extreme example being macrophages, which constantly sample the extracellular environment via this process (Lin et al., 2020). In endothelial cells, macropinocytosis occurs at a low background level but can be elevated in the presence of external stimuli, such as growth factors (Lin et al., 2020). By this process, endothelial cells take up materials from blood, including extracellular vesicles (Ajikumar et al., 2019) and platelets (Faille et al., 2012).

## Evidence for macropinocytosis of *Cn* by brain endothelial cells

Huang et al., (Huang et al., 2011) concluded that “*C. neoformans* may utilize the endocytic signaling pathway in [brain endothelial cells] to traverse the blood-brain barrier,” proposing that macropinocytosis was one of the potential types of endocytosis utilized by *Cn* but did not come to a final conclusion. Brain endothelia has been shown to engage macropinocytosis when exposed to viruses, bacteria, platelets and microvesicles (Liu et al., 2002; Faille et al., 2012; Loh et al., 2017; Ajikumar et al., 2019; Espinal et al., 2022). Evidence for *Cn* transcellular invasion of human brain endothelial cells beginning with internalization by macropinocytosis includes the dramatic plasma membrane rearrangement observed upon contact with *Cn*, including the formation of membrane projections that engulf fungal cells (Vu et al., 2013; Aaron et al., 2018). These rearrangements result from *Cn* inducing actin polymerization (profilin upregulation, cofilin downregulation) upon contact (Chen et al., 2003; Maruvada et al., 2012). Although this observation alone could also point to phagocytosis, the lack of identification of a specific receptor after decades of research is consistent with a nonspecific process. In other words, macropinocytosis represents a null hypothesis for *Cn* entry – if increasingly exhaustive screens fail to identify a ligand indicative of phagocytosis, the conclusion ought to default to *Cn* entry into brain endothelial cells relying on macropinocytosis.

There are other positive indications of macropinocytosis for *Cn* entry. Rac 1, commonly implicated in macropinocytosis of extremely diverse extracellular entities, is targeted by *Cryptococcus* (Maruvada et al., 2012). Other pathogens such as *E. coli* K1 (Loh et al., 2017) have also been shown to invade human brain endothelial cells in a Rac1-dependent macropinocytic pathway. Engulfment of *Cn* by brain endothelial cells is not a dead end for the pathogen as *Cn* can survive intracellularly *in vivo* (Davis et al., 2016; Chen et al., 2021). Heat killed *Cn* can adhere to the brain endothelium, but must be viable in order to be internalized and cross, implying that the process leading to adherence and internalization are separate, unlike what would be expected from an entirely receptor-mediated process (Shi et al., 2010; Chen et al., 2021). Disruption of actin filaments by cytochalasin D prevents *Cn* invasion of brain endothelial cells, demonstrating that entry is facilitated in an actin-dependent manner (Jong et al., 2008b).

## How macropinocytosis might be conclusively demonstrated for *Cn* in brain endothelial cells

Because macropinocytosis combines several features associated with both phagocytosis and pinocytosis, macropinocytosis can be difficult to establish experimentally. Endocytic vesicle size and membrane ruffling distinguish macropinocytosis from other forms of pinocytosis (Mercer and Helenius, 2009; Lin et al., 2020). High-resolution microscopy, preferably with actin staining to visualize the support structures beneath potential macropinosomes, is necessary to make this determination. However, protruding actin-supported membrane ruffles can superficially resemble phagocytosis of particles, but macropinocytosis and phagocytosis differ in that macropinocytosis also entraps a large extracellular fluid volume (Mercer and Helenius, 2009; Bloomfield and Kay, 2016). This can also be identified by microscopy, with staining for extracellular solutes or the introduction of large molecular weight tracer molecules (Espinal et al., 2022), or via flowcytometry (Loh et al., 2017). Fluorescently labelled dextran uptake can even be used quantitatively to determine the rate of endocytosis (Wang et al., 2014). Because both macropinocytosis, with its characteristic non-specific internalization of extracellular solutes, and BBB disruption would be expected to lead to increased blood solutes in the brain parenchyma, the presence or absence of these solutes cannot alone be used to differentiate between the pathways. Therefore, the presence and subcellular localization of tight-junction proteins must also be determined. If tight junctions are intact *in vivo* as is suggested *in vitro* (Chen et al., 2003), then macropinocytosis is indicated over paracellular leakage.

Macropinocytosis is also dependent on a number of processes that can be chemically inhibited; inhibition of actin polymerization, Rac1/Cdc42; and Na/H^+^ exchange should all also lead to a decrease of internalization (Mercer and Helenius, 2009). The most critical set of experiments for determining involvement of macropinocytosis in *Cn* CNS invasion is chemical inhibition of BBB crossing by amiloride (or one of its derivatives), accompanied by a lack of inhibition when alternate pathways are specifically blocked. Amiloride and its derivatives block macropinocytosis by preventing the dissipation of low submembranous pH, which inhibits the activity of GTPase activity crucial for actin polymerization (Koivusalo et al., 2010). Cholesterol is also critical for Rac1 membrane localization leading to macropinocytosis in at least some cells (Grimmer et al., 2002). By contrast, specific inhibitors of other forms of endocytosis, such as clathrin- and caveolin-dependent processes, should have little effect on *Cn* internalization (Medrano-Gonzalez et al., 2021). However, the outcome of each of these tests may not be completely binary, as macropinocytosis is by definition non-specific, and many pathogens are known to invade host cells through other endocytic pathways in parallel with macropinocytosis (Bauherr et al., 2020).

## Conclusions and future directions

As a pathogen, *Cn* is unusual in that its neurotropism drives its translocation from blood to brain despite the restricted passage imposed by the BBB; however, the reasons underlying the neurotropism are speculative in the absence of a BBB specific receptor/molecules. While EphA2 (and CD44) is not specific to brain endothelial cells, by engaging EphA2 *Cn* may be hedging its bets to avoid destruction in the bloodstream and instead exploit an EphA2-mediated binary pathway across the BBB into the CNS, where *Cn* can continue to thrive. This binary path involves both an EphA2-mediated macropinocytic transcellular pathway and a paracellular path via the re-modeling of the tight junctions by EphA2 (**Figure 1**). Additional work to investigate the molecular and cellular interplay between *Cn*, EphA2, its downstream signaling components and the NVU in mediating the translocation of *Cn* from blood-to-brain will be necessary to test this idea. The commonality among diverse pathogens to co-opt EphA2 in order to access the host is an emerging area of study that may yield a therapeutic intervention with broad spectrum activity.

## Author contributions

DL: Conceptualization, Writing – original draft, Writing – review & editing. AB: Conceptualization, Writing – original draft, Writing – review & editing. KV: Conceptualization, Writing – original draft, Writing – review & editing. AG: Conceptualization, Funding acquisition, Supervision, Writing – original draft, Writing – review & editing.
